# MRPL12 regulates high glucose-induced ferroptosis in renal tubular epithelial cells via GPX4

**DOI:** 10.1016/j.bbadva.2026.100197

**Published:** 2026-06-23

**Authors:** Guoying Xia, Weiwei Zhang, Jinshi Liu, Jian Yang, Qiuyao Jiang, Wei Xin

**Affiliations:** a Department of Clinical Laboratory, Shandong Provincial Key Laboratory of Preventionand Treatment of Major Chronic Diseases in Medicine and Health, The Third Affiliated Hospital of Shandong First Medical University (Affiliated Hospital of Shandong Academy of Medical Sciences) , Jinan, China; b College of Basic Medicine, Henan University, Kaifeng, Henan, China; c Central Laboratory, Shandong Provincial Hospital Affiliated to Shandong First Medical University, Jinan, China; d Medical Science and Technology Innovation Center, Shandong First Medical University & Shandong Academy of Medical Sciences, Jinan, China

**Keywords:** MRPL12, Ferroptosis, DKD, GPX4, P53, MDM2

## Abstract

Diabetic kidney disease (DKD) is a severe chronic complication of diabetes and one of the major causes of end-stage renal disease (ESRD). Increasing evidence implicates ferroptosis in pathological cell death associated with metabolic diseases, carcinogenesis, and ischemia-reperfusion injury, which is also thought to be involved in kidney tubular cell death under diabetic conditions; however, the underlying pathogenic mechanism remains unclear. We found increased iron level and cellular lipid peroxidation in streptozotocin (STZ)-induced DKD mice and high glucose-stimulated renal tubular cells, accompanied by mitochondrial morphological and functional damages. Further research showed that the expression of mitochondrial ribosomal protein L12 (MRPL12) was downregulated in high glucose conditions. Overexpression of MRPL12 alleviated the damages caused by high glucose-induced ferroptosis in renal tubular cells. MRPL12 interacts with murine double minute 2 (MDM2), an E3 ubiquitin-protein ligase involved in the ubiquitination degradation pathway of several proteins. MRPL12 plays a crucial regulatory role in high glucose-induced ferroptosis of renal tubular epithelial cells by modulating glutathione peroxidase 4 (GPX4), which is a potential novel therapeutic target in improving mitochondrial homeostasis and promoting renal function.

## Highlights

• MRPL12 plays a crucial regulatory role in high glucose-induced ferroptosis of renal tubular epithelial cells by modulating GPX4.
• GPX4 is a direct target gene of p53.
• The upregulation of MRPL12 alleviates diabetic renal injury, and inhibits the process of ferroptosis in DKD.

## Graphical abstract

**Figure:**
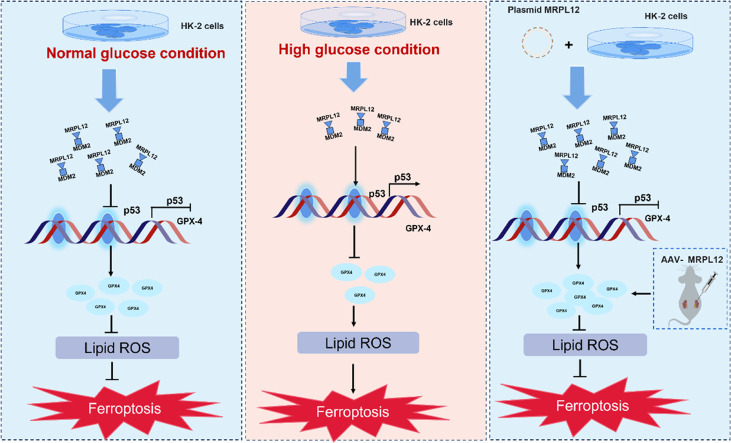

## 1 Introduction

Diabetic kidney disease (DKD), a serious microvascular complication of diabetes, is a leading cause of chronic kidney disease (CKD) and ESRD, which are characterized by proteinuria and a progressive decline in kidney function [1,2]. International studies indicate that the incidence of ESRD in patients with diabetes is approximately 10 per 1000 person-years. Among those with macroalbuminuria, the ESRD incidence rises to nearly 60 per 1000 person-years [3]. Approximately 20–40% of diabetic patients develop DKD [4]. The primary clinical goal for current DKD treatment is to relieve symptoms and delay disease progression, although treatment efficacy remains limited [5]. Therefore, understanding the pathophysiological mechanism of DKD and identifying potential intervention targets is crucial. Conventional viewpoints indicate that hyperglycemia, hemodynamic changes, and local growth factors are all involved in the pathogenesis of DKD [6,7].

Recent studies have reported that ferroptosis is involved in DKD [[8], [9], [10]], but the precise mechanism is unclear. Ferroptosis is an iron-dependent form of regulated cell death, characterized by the accumulation of lipid peroxides, and requires abundant cellular iron [[11], [12], [13]]. The accumulation of polyunsaturated phospholipid hydroperoxides in the presence of ferrous ions leads to rapid membrane rupture and irreversible cell death [14,15]. Lipid reactive oxygen species (ROS), which are essential for ferroptosis, are primarily generated as a byproducts of cellular metabolism.

Several organelles are associated with multiple ferroptosis pathways, including mitochondria, lysosomes, Golgi apparatus, and endoplasmic reticulum. Transmission electron microscopy (TEM) analysis showed a striking morphological change in mitochondria following DKD, characterized by reduced volume and increased membrane density. Mitochondria play an important role in the regulation of cellular metabolism and death signaling. MRPL12 was the first mitochondrial ribosomal subunit cloned in humans, and was identified based on positive regulation of its mRNA expression by growth factors. It is encoded by nuclear genes, synthesized in the cytoplasm, and imported into mitochondria. MRPL12 is involved in homeostatic regulation of mitochondrial transcription and ribosome biogenesis that likely contribute to cell cycle, growth regulation, and longevity pathways. In addition, as a transcription factor, MRPL12 can bind directly to mitochondrial RNA polymerase (POLRMT) and activate the transcription of mtDNA. We recently found that high glucose treatment causes differential expression of MRPL12 in human renal proximal tubular epithelial cells (HK-2) [16]. However, the precise role of MRPL12 in DKD has not been reported yet.

GPX4, as a key regulator of ferroptosis, maintains membrane stability by reducing lipid peroxides. Research data demonstrate a significant association between the functional status of GPX4 and the pathological progression of renal injury. In DKD, the hyperglycemic environment downregulates GPX4 expression by suppressing the Nrf2 pathway, leading to mitochondrial ROS accumulation and lipid peroxidation, which consequently damages podocytes and renal tubular epithelial cells. Moreover, GPX4 deficiency exacerbates glomerular basement membrane thickening and mesangial expansion, promoting proteinuria formation. Notably, deficiency of MRPL12, a core mitochondrial translation component, impairs oxidative phosphorylation and elevates ROS production, ultimately inducing apoptosis in renal tubular epithelial cells. Additionally, studies have revealed that the mitochondrial enzyme DHODH protects against mitochondrial lipid peroxidation by regenerating ubiquinol (the reduced form of coenzyme Q10). Meanwhile, GPX4 exhibits mitochondrial localization and can eliminate mitochondrial lipid peroxides. However, whether there is any functional interplay between these two mechanisms remains unclear.

In the present study, we found that the expression of MRPL12 is decreased in DKD and that MRPL12 regulates the occurrence of ferroptosis. Furthermore, we identified that MRPL12 overexpression could alleviate long-term high glucose-induced renal damage. Specifically, we found that MRPL12 regulates the expression of GPX4 via interaction with murine double minute 2 (MDM2), which subsequently inhibits p53, a transcription factor of GPX4. The interaction of MRPL12 with MDM2 is potentially a novel therapeutic target for improving mitochondrial homeostasis and function.

## 2 Materials and methods

### 2.1 Cell lines and cell treatments

HK-2 cells were obtained from the American Type Culture Collection (Rockville, MD, USA). Cells were cultured in Dulbecco's Modified Eagle Medium ((5.5 mM)) (DMEM; Gibco™, Grand Island, NY, USA) containing 10% fetal bovine serum (FBS; Gibco® Grand Island, NY, USA) and 1% penicillin-streptomycin, in a humidified atmosphere of 5% CO_2_ at 37 °C. Cells were transfected with siRNAs targeting MRPL12 and GPX-4 using riboFECT™CP Transfection kit (RiboBio) or MRPL12 overexpression plasmid using Lipofectamine 3000 (Invitrogen) according to the manufacturers’ instructions. MRPL12-OE and MRPL12-KD denote the overexpression and knockdown of MRPL12, respectively. For high-glucose treatment, we supplemented the low-glucose culture medium with glucose (Solarbio, G8150) to a final concentration of 30 mM. Meanwhile, mannitol (Solarbio, M8140)was used as an osmotic control to match the osmolarity of the high-glucose medium.

### 2.2 Cell viability

Cell Counting Kit 8 (CCK-8; Dojindo, Kumamoto, Japan) was used to measure cell viability. To determine cell viability, 10 μL of CCK-8 solution was added to each well of the 96-well plate containing treated and control samples, and incubated for 2 h at 37 °C. All the experiments were performed three times, and cell viability was measured at 450 nm.

### 2.3 Prussian blue iron staining

Following standard dewaxing and rehydration of paraffin-embedded mouse kidney tissue sections, Perls working solution was applied to the sections for iron staining (15–30 min incubation at room temperature), followed by a 5-min distilled water rinse. Nuclear counterstaining was then performed by covering the sections with nuclear fast red solution for 10 min, with a brief 5-second distilled water wash. After completing routine dehydration through an ethanol series and xylene clearing, the sections were mounted with neutral balsam for microscopic examination. Prussian blue-positive iron deposits (blue) and nuclear staining (red) were evaluated under light microscopy.

### 2.4 Immunohistochemistry

Immunohistochemistry staining was performed using *An sp* Kit (SP-9001; Zhongshan Golden Bridge) according to the manufacturer’s protocol, kidney sections (5-μm-thick, paraffin-embedded) were incubated with anti-4-HNE primary antibodies overnight at 4 °C, and the slides were then observed under a Nikon microscope imaging system (Nikon Ti-S, Tokyo, Japan).

### 2.5 FeRhoNoxTM-1 fluorescent probes staining

A 1 mM FeRhoNox™-1 stock solution was diluted in 1× phosphate buffered saline (PBS) to prepare a 10 μM working solution. The cell culture medium was removed, and the working solution was added to completely cover the cells. After incubation at room temperature for 3 min, the working solution was discarded, and the cells were washed three times with PBS. The cells were then filtered and analyzed using flow cytometry.

### 2.6 Lipid peroxides measurement

To visualize the lipid peroxides, cells were seeded in 6‐well plates and treated under specific conditions. Subsequently, the cells were stained with 5 μM C11-BODIPY581/591 probe (D3861; Molecular Probes, USA) in accordance with the manufacturer's instructions. After incubation at 37 °C for 30 min, the cells were stained with Hoechst nuclear stain (Dojindo) for 20 min. Then, the cells were washed with PBS and observed using a Leica SP8 confocal laser scanning microscope (Leica Microsystems). The fluorescence intensity was quantified using ImageJ software. The mean fluorescent intensity for each group was normalized to that of the control group. C11-BODIPY581/591 fluorescence was analyzed using fluorescence-activated cell sorting (FACS) (FACScan; BD Biosciences, San Jose, *CA*, USA) and then evaluated using the FlowJo 7.6 software. Data were collected from at least 10,000 cells.

### 2.7 Detection of intracellular reactive oxygen species (ROS)

Intracellular ROS was detected via DCFH-DA staining. After treatment, cells were incubated with 10 μM DCFH-DA in serum-free medium at 37 °C for 25 min in dark. Cells were rinsed with PBS and counterstained with DAPI. Fluorescence images were obtained under fluorescence microscope, and relative ROS intensity was quantified by ImageJ software.

### 2.8 Transmission electron microscope (TEM)

Multiple 1 mm³ tissue blocks were excised from the renal cortex using surgical scissors and immediately fixed in ice-cold 2.5% glutaraldehyde solution (4 °C) for optimal ultrastructural preservation. Following fixation, tissues were progressively dehydrated through a graded acetone series (50%, 70%, 90%, and 100%). The samples were then embedded in epoxy resin and sectioned into 70nm ultrathin slices using an ultramicrotome. For enhanced contrast, sections were double-stained with uranyl acetate (2% aqueous solution) and lead citrate (Reynolds' formulation). Ultrastructural examination was subsequently performed using a JEM-100sX transmission electron microscope.

### 2.9 Measuring ATP content

The ATP content in cells was measured using intracellular ATP assay kit (S0026; Beyotime). First, 100 µl of ATP assay working solution was added to the assay wells and left at room temperature for 5 min to deplete the background ATP. Then, 20 µl of sample or standard was added to the assay wells. After an interval of at least 2 s, the relative light units (RLU) value or counts per minute (CPM) was determined with chemiluminescence instrumentation. ATP concentration was normalized to protein concentration.

### 2.10 Quantification of mitochondrial DNA copy numbers

The total DNA was extracted with a genomic DNA isolation kit (No. D0063; Beyotime). For determining mtDNA copy number, total DNA was isolated as abovementioned, and quantified with qPCR using short mtDNA primers to measure mtDNA content. G6PC primers were used as the nuclear control to normalize mitochondrial to nuclear gene ratio. The primer sequences were as following:

G6PC: F-CTGTCTTTGATTCCTGCCTCAT; R-GTGGCTGTGCAGACATTCAA. D-Loop2: F-GGCTCTCAACTCCAGCATGT; R-AGGACGAGGGAGGCTACAAT.

### 2.11 Mitochondrial fluorescent staining

For MitoTracker Red CMXRos staining, 50 µg of MitoTracker Red CMXRos powder (M7512; Thermo Fisher Scientific) was dissolved in 470 µL of dimethyl sulfoxide (DMSO) (D8371; Solarbio) to prepare a 200 µM stock solution. A small aliquot of the stock solution was then diluted 1:1000 in cell culture medium to obtain a 200 nM working solution. The cells were cultured in dishes for the required duration, and incubated with 200 nM working solution at 37 °C for 30 min. For MitoSOX Red staining, a 5 mM stock of MitoSOX Red (MSR) reagent was prepared by dissolving the contents of the vial in anhydrous DMSO, which was then diluted with culture medium to prepare a 500 nM working solution. The cells were incubated for 30 min at 37 °C and 5% CO_2_, and gently washed 3 times with warm PBS. Finally, the stained cells were observed or detected using a fluorescence microscope.

### 2.12 Quantification of mRNA using quantitative real-time PCR

Total RNA was extracted from treated cells using TRIZoL Reagent (No.15596026; Invitrogen). mRNA was reversed transcribed into cDNA with the PrimeScript RT reagent kit (DRR047A; TAKARA) in a 20 μL reaction system. The PCR reaction was carried out on a Roche LightCycle 480 (Roche Applied Sciences, Mannheim, Germany) under the following thermal cycling conditions: 95  °C for 30 s, 40 cycles of 95  °C for 5 s, and 60  °C for 30 s. Each target gene expression was normalized to that of β-actin.

### 2.13 Western blotting (WB)

Treated cells were harvested at the indicated points and lysed in radio-immunoprecipitation assay (RIPA) buffer containing a protease inhibitor and phosphatase inhibitor cocktail. Proteins were separated with 12.5% sodium dodecyl sulfate-polyacrylamide gel electrophoresis (SDS-PAGE) and transferred onto a polyvinylidene fluoride (PVDF) membrane (Millipore). Membranes were blocked with 5% non-fat milk at room temperature for 1 h, then incubated in primary antibodies overnight at 4 °C. Primary antibodies were detected with horseradish peroxidase (HRP)-conjugated goat anti-rabbit secondary antibody and visualized with Storm 680 phosphorimager (Molecular Devices). The images were analyzed using Image J software.

### 2.14 Immunofluorescence staining

Briefly, HK-2 cells were seeded in chamber slides, treated for 72 h, and fixed with 4% paraformaldehyde. After washing 3 times for 5 min with PBS, cells were permeabilized in 0.1% Triton X-100 and blocked for 1 h with 1% goat serum (SL038; Solarbio), and incubated with a GPX4 primary antibody (1:300; ab62352; Abcam) and MRPL12 primary antibody (1:200, 14,795-1-AP; Proteintech) overnight at 4 °C. This was followed by 1 h incubation at 37 °C with secondary antibody Alexa Fluor®−488 and secondary antibody Alexa Fluor®−594 (1:1000, Invitrogen). After incubation, the nucleus was counterstained with Hoechst 33,342 (H3570; Life technologies). The slides were mounted with fluorescent mounting medium (Dako). Fluorescent images were obtained with Nikon microscope imaging system (Nikon Ti-S, Tokyo, Japan) and analyzed using Image J software.

### 2.15 Malondialdehyde (MDA)

The MDA content was measured following manufacturer's protocol (ab118970), and the absorbance at 532 nm was measured using a microplate reader (Thermo, USA).

### 2.16 Chromatin immunoprecipitation (ChIP) analysis

To separate the chromatin, cells were lysed using the ChIP analysis kit (No. 10,086; Merck) according to the manufacturer’s instructions, and sonicated. DNA was immunoprecipitated from the sonicated cell lysates using 2 μg p53 antibody (Cat#10,442-1-AP; Proteintech), 1 μg RNA polymerase II antibody (Magna ChIP A/G) and 1 μg normal mouse IgG antibody (Magna ChIP A/G). Finally, primers were used for standard end-point PCR and real-time quantitative PCR. The primer sequence contained the binding site of p53 and GPX4 promoter predicted by ALLGEN PROMO3.0.2, the ChIP Atlas, Cistrome DB, and hTFtarget.

### 2.17 Co-immunoprecipitation (Co-IP)

We verified protein-protein interactions using the Pierce™ Crosslink IP Kit (Cat# 26,147; Thermo Scientific) following the manufacturer's protocol. For forward Co-IP, MRPL12 lysates were immunoprecipitated with anti-MRPL12 antibody, followed by immunoblotting with anti-MDM2 antibody. Conversely, reverse Co-IP was performed by precipitating MDM2 with its specific antibody and probing for MRPL12. All antibodies were validated for specificity.

### 2.18 Mouse models

Six-week-old male C57BL/6 J mice were purchased from Shandong University Experimental Animal Center. We housed all mice on a 12-h light-dark cycle and provided them with free access to food and water for 13 weeks. All experiments were approved by Animal Experimental Ethical Inspection of Shandong Provincial Hospital. A total of 45 mice were randomly divided into four groups as follows: normal control mice (non-DKD group, n = 10), streptozotocin (STZ)-induced DKD mice (DKD group, n = 10), AAV-MRPL12 mice (AAV-MRPL12 group, n = 10), and AAV-MRPL12 DKD mice (AAV-MRPL12 DKD group, n = 15). AAV2/9-ctr.sh or AAV2/9-HU6-shMPL12 was injected bilaterally into the kidney in 25 mice, as described above, and the mice were allowed to recover for a week. The DKD groups were fasted for12 h and injected with 50 mg/kg STZ intraperitoneally on5consecutive days. Control animals received the same volume per body weight of citrate buffer. Blood glucose levels of the mice were measured using blood glucose test strips after a week of STZ injection, and blood glucose levels > 16.7 mM were defined as diabetes; mice with random blood glucose levels < 16.7 mM were excluded from the experiment.

### 2.19 Hematoxylin and eosin (H&E) staining and periodic acid schiff (PAS) staining

Briefly, the kidney was fixed with 4% paraformaldehyde overnight at room temperature, and then embedded in paraffin. The tissue was sliced into 5 µm-thick slices. The tissue was stained at room temperature with H&E staining (G1120, Solarbio) and PAS staining (G1281, Solarbio). The slide was observed under a microscope (Nikon Ti-S, Tokyo, Japan).

### 2.20 Renal function measurement

Urine was collected in metabolic cages and centrifuged at 3000 rpm for 15 min at 4 °C. Urine protein level and β2 microglobulin level were assessed using commercial kits (Nanjing Jiancheng Bioengineering Institute, Nanjing, China) according to the manufacturer's instructions.

### 2.21 Statistical analysis

Statistical data are expressed as mean ± standard deviation (SD). Each experiment was performed at least three times, and the data were statistically analyzed in GraphPad Prism 8.0 (GraphPad Software) using *t*-tests or one-way analysis of variance (ANOVA). For two groups comparison, the Student’s *t*-test was used with homogeneity of variance, and the *t*-test was used with heterogeneity of variance. For multiple groups comparison, one-way ANOVA was used with homogeneity of variance and the Welch method was used with heterogeneity of variance. Statistical significance was set at P values < 0.05. *P < 0.05, **P < 0.01, ***P < 0.001.

## 3 Results

### 3.1 High glucose-induced HK-2 cells and the kidney of DKD mice exhibit high glucose-induced ferroptosis

**Fig. 1:**
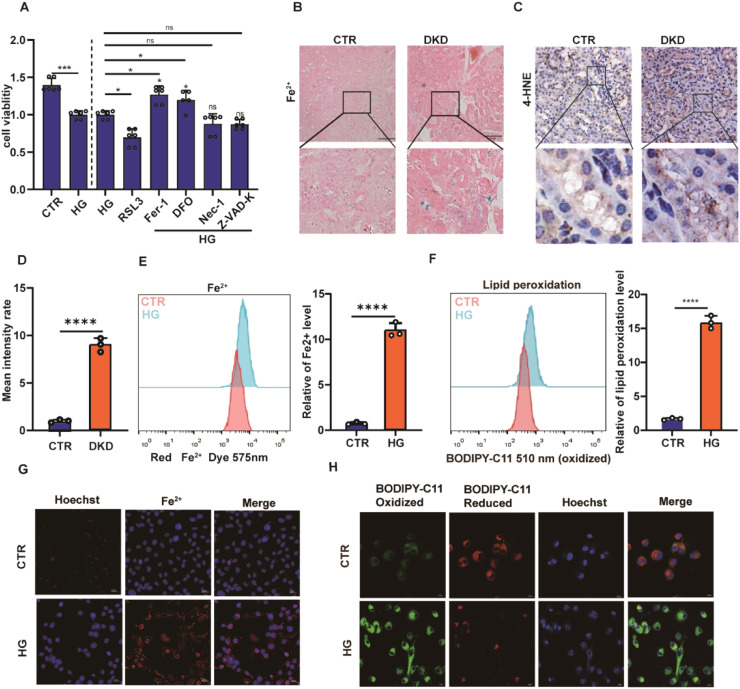
High **glucose-induced HK-2 cells and the kidney of DKD mice exhibit high glucose-induced ferroptosis. (A)** Relative viability of HK2 cells treated separately with 30 mM glucose and with Fer-1 (5 μm), DFO (5 μm), Ner-1 (5 μm), and Z-VAD-K (5 μm) for 72 h. n = 6, *P < 0.05, **P < 0.01, ***P < 0.001. **(B)** Representative Prussian blue staining images showed renal iron content in mice. The scale bars are 100 μm. **(C)** Dark brown-stained cells indicate 4-HNE-positive staining; scale bars, 50 μm. **(D)** Bar graphs showing the percentage of 4-HNE-positive tubules per visual field. **(E)** HK-2 cells were treated with high glucose (30 mM) for 72 h. Measurement and quantification of intracellular Fe^2+^ levels with flow cytometry using FeRhoNoxTM-1 fluorescent probes. **(F)** Measurement and quantification of intracellular lipid peroxidation levels with flow cytometry using BODIPY-C11 fluorescent probes in high glucose-treated HK-2 cells. **(G)** Fluorescent microscope images of Fe^2+^ in HK-2 cells treated with 30 mM glucose for 72 h. Representative images of experiments repeated three times. **(H)** Lipid peroxidation was measured with BODIPY at 493/503 nm using Immunofluorescent staining in high glucose-induced HK-2 cells. Scale bar, 25 µm. Representative images of experiments repeated three times. *P < 0.05, **P < 0.01, ***P < 0.001.

Cells were treated with ferroptosis inhibitor (Fer-1), iron chelator (DFO), ferroptosis activators (RSL3), caspase inhibitor (Z-VAD-K), and necroptosis inhibitor (Nec-1) under high glucose condition. The results revealed that Fer-1 and DFO alleviated cell damage from high glucose, while RSL3 exacerbated cell damage, indicating that ferroptosis plays a vital role in high glucose-treated renal tubular cells (Fig. 1A). The level of Fe^2+^ and 4-HNE were increased in kidney tissues in DKD mice compared to normal control (Fig. 1B-1D). Ferroptosis levels were estimated using immunofluorescent staining and flow cytometry (FCM) assay to investigate iron level and cellular peroxidation, which showed significantly increased Fe^2+^and lipid peroxidation (Fig. 1E-1H). These results suggest that ferroptosis may be involved in the pathogenesis of DKD and high glucose-treated cells.

### 3.2 High glucose decreases MRPL12 and aggravates mitochondrial damages

**Fig. 2:**
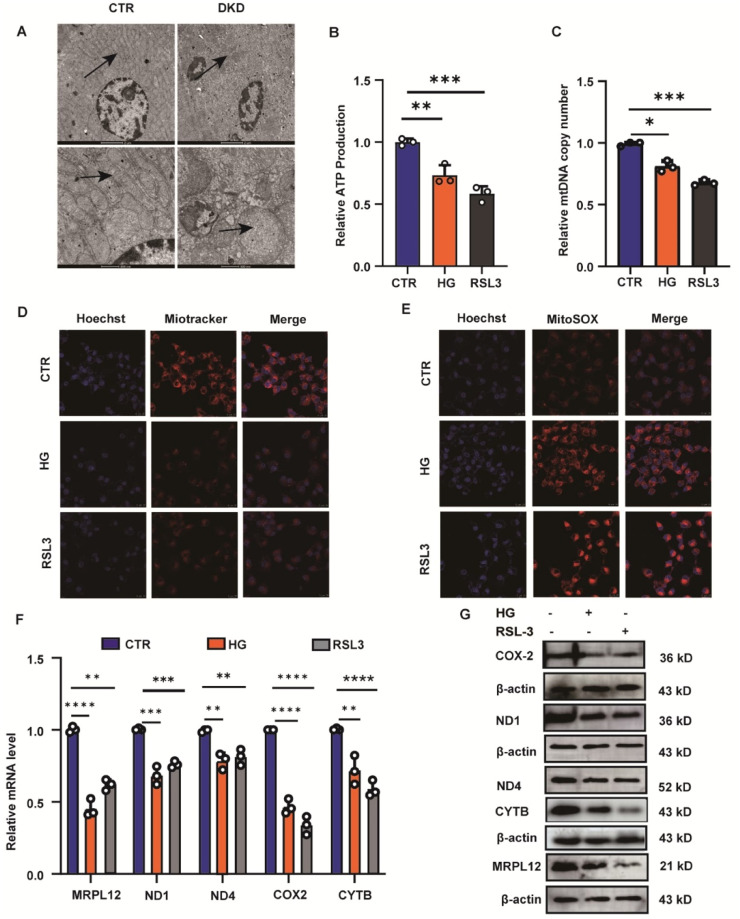
High glucose **decreases MRPL12 and aggravates mitochondrial damages. (A)** TEM analysis for TECs mitochondria in renal samples of WT and DKD mice. Scale bars, 2 μm. **(B)** ATP production from HK-2 cells exposed to high glucose (30 mM). **(C)** mtDNA copy number in HK-2 cells treated with high glucose (30 mM). G6PC were used as the nuclear control to normalize mitochondrial to nuclear gene ratio. n = 3 per group, *P < 0.05, **P < 0.01, ***P < 0.001. **(D-E)** Immunofluorescent staining on high glucose and RLS-3 treated HK-2 cells with Mito Tracker Red and Mito-Sox separately. Scale bar, 20 µm. **(F)** Levels of the mt DNA-coded OXPHOS components and MRPL12 mRNAs in HK-2 cells treated with high glucose and RSL-3 for 72 h were analyzed using RT-qPCR. Relative expression levels were normalized to the β-actin expression levels. **(G)** Protein levels of the mtDNA-coded OXPHOS components (CI: ND1, ND4; CIII: CYTB; CIV: COX-2:) and MRPL12 determined by western blotting in HK-2 cells treated with high glucose and RSL-3 for 72 h. Data are the means ± SD (n = 3 independent experiments) *P < 0.05, **P < 0.01, ***P < 0.001.

Ferroptosis is closely related to mitochondrial damage. TEM analysis showed mitochondrial injuries In the kidneys of DKD mice, such as morphologic changes characterized by reduced volume, increased membrane density, and cristae loss (Fig. 2A). Based on changes in mitochondrial morphology, we stimulated HK-2 cells with high glucose or ferroptosis inducer RSL3 that resulted in significantly decreased ATP production (Fig. 2B), and reduced mtDNA copy number (Fig. 2C) and mitochondrial abundance (Fig. 2D), along with increased mitochondrial ROS generation (Fig. 2E). At the molecular level, we assessed the expression and activity of mitochondrial respiratory chain complex subunits ND1(NADH-ubiquinone oxidoreductase chain 1), ND4 (NADH dehydrogenase subunit 4), cytochrome b (CYTB), cytochrome c oxidase subunit II (COX-2)) using WB and RT-qPCR and found that the mRNA (Fig. 2F) and protein levels (Fig. 2G) of ND1, ND4, CYTB, and COX-2 in HK-2 cells decreased. This demonstrates that ferroptosis and DKD (or high glucose) consistently induce damage to both mitochondrial morphology and function. MRPL12 is a constituent protein of the mitochondrial ribosome large subunit, belonging to the mitochondrial ribosomal protein (MRP) family. It plays a crucial role in mitochondrial protein translation by participating in the synthesis of mitochondrial DNA (mtDNA)-encoded proteins, thereby influencing mitochondrial function, energy metabolism, and cellular homeostasis [16]. High glucose treatment decreased mRNA levels of *MRPL12* (Fig. 2F); furthermore, WB analysis confirmed high glucose-induced changes in MRPL12 expression (Fig. 2G). Taken together, the results show that mitochondrial morphology and function were damaged in DKD and high glucose-induced HK-2 cells.

### 3.3 MRPL12 is involved in the regulation of high glucose-induced ferroptosis

**Fig. 3:**
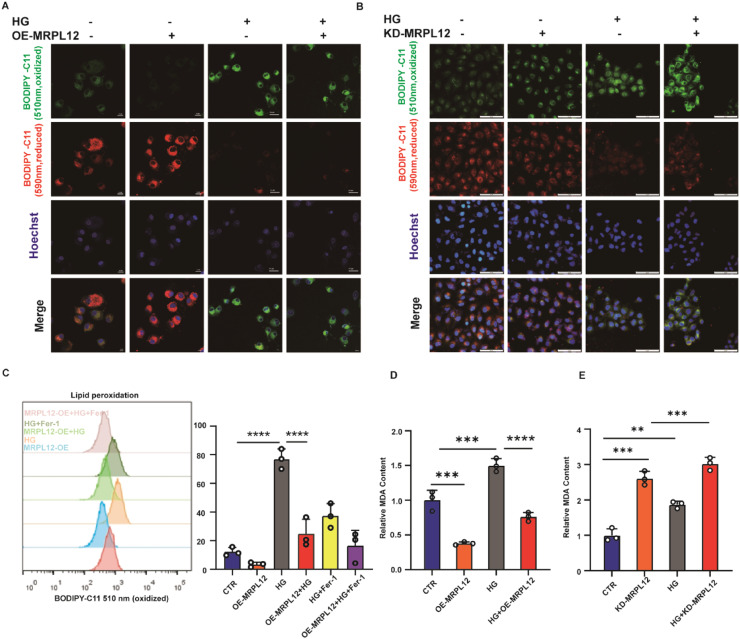
MRPL12 is **involved in regulating high glucose-induced ferroptosis. (A-B)** Fluorescent microscope images of BODIPY-C11 in MRPL12 overexpressing and knockdown HK-2 cells. Scale bar, 25 µm. Representative images of experiments repeated three times. **(C)** Flow cytometry analysis of lipid peroxidation levels reported by BODIPY-C11 in MRPL12 overexpressing HK-2 cells treated with 30 mM glucose for 72 h. Representative plots of experiments repeated three times. **(D-E)** Analysis of MDA levels in MRPL12 overexpressing and knockdown HK-2 cells following high glucose treatment for 72 h (n = 3, *P < 0.05).

In this study, we hypothesized that MRPL12 can protect against high glucose-induced ferroptosis in HK-2 cells. Immunofluorescence staining revealed that MRPL12 overexpression mitigated the level of intracellular lipid peroxidation caused by high glucose (Figs. 3A, C, D). Flow cytometry analysis showed that MRPL12 overexpression decreased high glucose-induced lipid peroxidation. MDA is one of the end products of the lipid peroxidation reaction. MDA analysis was also used to test lipid peroxidation, and the results indicated that MRPL12 overexpression reduced the end products of lipid peroxidation induced by high glucose treatment. Additionally, MRPL12 knockdown obviously aggravated lipid peroxidation (Fig. 3B) and increased high glucose-induced MDA levels (Fig. 3E). The abovementioned results all suggest that MRPL12 can alleviate the degree of ferroptosis, reduce lipid peroxidation, and decrease MDA production in high glucose-treated HK-2 cells.

## 4 MRPL12 regulates ferroptosis via GPX4 signaling pathway

**Fig. 4:**
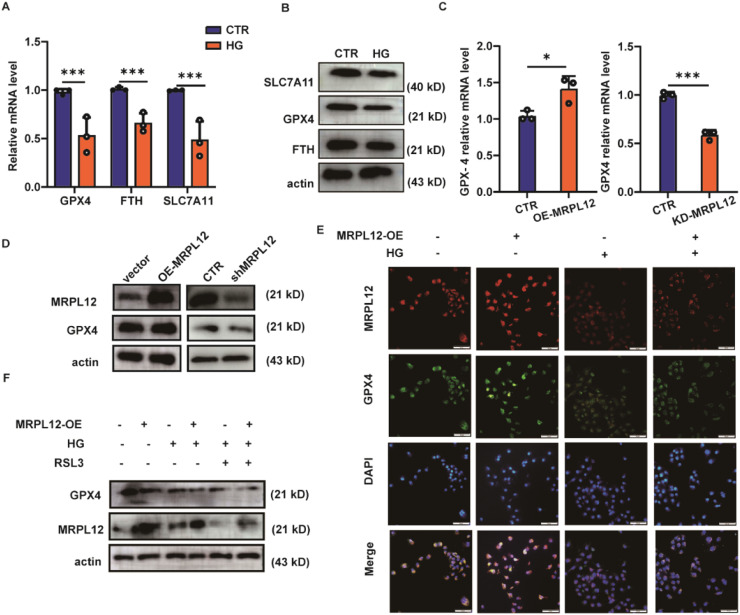
**MRPL12 regulates ferroptosis via GPX4 signaling pathway. (A)** Levels of GPX4, FTH, and SLC7A11 mRNA in high glucose-induced HK-2 cells were analyzed using RT-qPCR. Relative expression levels were normalized to β-actin expression levels. Data are expressed as means ± SD (n = 3 independent experiments). **(B)** Western blotting analysis showing the levels of GPX4, FTH, and SLC7A11 proteins in HK-2 cells under high glucose condition. **(C)** Levels of GPX4 in MRPL12-knockdown and overexpressing HK-2 cells were analyzed using RT-qPCR. Relative expression levels were normalized to β-actin expression levels. Data are shown as means ± SD (n = 3 independent experiments). *P < 0.05, **P < 0.01,***P < 0.001. **(D)** Western blotting showing the levels of GPX4 and MRPL12 protein expression in MRPL12-knockdown and overexpressing HK-2 cells. Data are presented as mean ± SD (n = 3). *P < 0.05. **(E)** Immunofluorescent staining was performed to detect MRPL12 and GPX4 protein expression in overexpressing HK-2 cells under high glucose condition. **(F)** Western blotting showing the levels of GPX4, protein expression in MRPL12-overexpressing HK-2 cells in response to high glucose (30 mM, 72 h), RSL3 (5 µM, 24 h). Data are presented as means ± SD (n = 3). *P < 0.05, **P < 0.01, ***P < 0.001.

We continued to explore the possible underlying roles of MRPL12 in ferroptosis. First, the mRNA levels of ferroptosis markers, such as GPX4, solute carrier family 7 member 11 (SLC7A11), and ferritin heavy chain (FTH), were reduced in high glucose-induced HK-2 cells (Fig. 4A). Similarly, the protein expression of GPX4, SLC7A11, and FTH was reduced upon high glucose treatment (Fig. 4B). MRPL12 knockout led to a significant reduction in GPX4 expression at both protein and mRNA levels. Conversely, MRPL2 overexpression resulted in elevated levels of GPX4 at both mRNA and protein levels (Fig. 4C-4D). The expression level of GPX4 was also elevated, as shown by immunofluorescent staining, when MRPL12 was overexpressed in high glucose-treated HK-2 cells (Fig. 4E). Finally, We overexpressed MRPL12 using a plasmid,and applied RSL3 as a positive control tfor ferroptosis.;and the results showed that the MRPL12-induced increase in GPX4 was blocked by RSL3 treatment (Fig. 4F). Following GPX4 knockdown, cellular lipid peroxidation levels were significantly increased(Fig.S2). In conclusion, GPX4 is involved in MRPL12-regulated ferroptosis.

## 5 MRPL12 regulates GPX4 transcription via interaction with MDM2

**Fig. 5:**
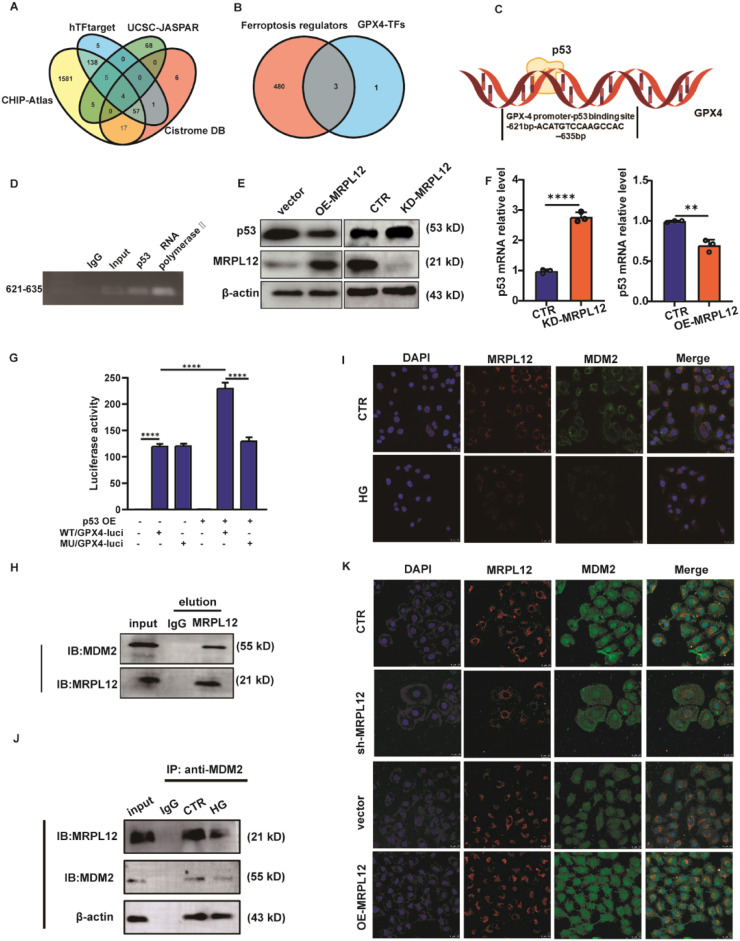
**MRPL12 regulates GPX4 transcription via interaction with MDM2. (A)** Upset-plot of Venn diagram of potential GPX4 transcription factors predicted by varied databases. **(B)** Venn diagram of potential transcription factors between ferroptosis regulators and GPX4. **(C)** The potential binding site of p53 within GPX-4 promoter region was predicted using ALGGEN-PROMO (version 3.0.2). **(D)** ChIP-PCR analysis of p53 binding to GPX4 promoter on HK-2 cells chromatin using rabbit anti-p53 antibody and rabbit IgG as negative controls. **(E)** Western blot analysis of MRPL12 and p53 in HK-2 cells in MRPL12-knockdown and overexpressing HK-2 cells. **(F)** mRNA level of p53 in HK-2 cells in MRPL12-knockdown and overexpressing HK-2 cells. **(G)** P53 overexpression significantly increased the GPX-4 luciferase reporter activity. **(H)** Immunoprecipitation demonstrates that MRPL12 interacts with MDM2. **(I)** Immunofluorescent staining was performed to detect MRPL12 and MDM2 protein expression in high-glucosed HK-2 cells. **(J)** Co-immunoprecipitation demonstrates that MDM2 interacts with MRPL12 in HK-2 cells under high-glucose conditions. **(K)** Immunofluorescent staining was performed to detect MRPL12 and MDM2 protein expression in MRPL12-knockdown and overexpressing HK-2 cells. *P < 0.05, **P < 0.01, ***P < 0.001.

We further explored the underlying mechanisms by which MRPL12 regulates the expression of GPX4. ChIP Atlas, Cistrome DB, ALGGEN PROMO, and hTFtarget were used to predict possible transcription factors of GPX4. The upset-plot of the Venn diagram from the four databases revealed a total of four possible transcription factors: p53, vitamin D receptor (VDR), androgen receptor (AR), and upstream stimulatory factor 2 (USF2) (Fig. 5A). Next, we utilized the ferroptosis regulators to predict possible co-transcription factors of the GPX4-transcriptional factors (TFs). The intersection of the three predicted TFs showed that p53, VDR, and AR may play a role in transcriptional regulation of GPX4 (Fig. 5B). Cyclin dependent kinase inhibitor 1A (CDKN1A) expression mediated by p53 delays the occurrence of ferroptosis in cancer cells in response to subsequent cystine deprivation. Considering that GPX4 might be regulated by p53 at the transcriptional level, Genome Browser and Jaspar were used to identify putative transcription factor binding sites within the GPX4 promoter region, which showed that the predicted promoter binding site ranged from 621 to 635 bp of GPX4. Then, the potential binding site was confirmed using ChIP-qPCR, and the core region was mapped to the sequence between −621 bp and −635 bp relative to the transcription start site (Fig. 5D).

MRPL12 overexpression led to a significant reduction in p53 at both protein and mRNA levels. Conversely, MRPL12 knockout resulted in elevated p53 levels for both the protein and mRNA (Fig. 5E-5F). The wild-type and mutant reporter gene plasmids of GPX4 promoter were constructed based on the binding site information of p53 and the promoter region of GPX4, respectively. The luciferase activity of 293T cells transfected with wildtype GPX4 reporter plasmid clearly increased after p53 overexpression, whereas that of cells transfected with the mutated GPX4 reporter plasmid was not increased (Fig. 5G). MDM2, an E3 ubiquitin-protein ligase involved in the ubiquitination degradation pathway of several proteins is known to inhibit p53-mediated transcriptional activity. MDM2 plays an important role in mitochondria bioenergetics [17]. In view of the role that MRPL12 plays in mitochondrial energy metabolism, we verified the interaction of MDM2 and MRPL12 using Co-IP assay. Input included the raw sample without immunoprecipitation, which contained the total protein. The results showed that MRPL12 can interact with MDM2 (Fig. 5H, 5J). MRPL12-MDM2 binding is reduced under high glucose or DKD conditions (Fig. 5I, 5 J). When observed under fluorescence microscope, the fluorescence signals of MDM2 and MRPL12 showed significant overlap in the cytoplasm (Fig. 5K). Collectively, these data indicate that GPX4 is a direct target gene of p53 and MRPL12 can interact with MDM2.

## 6 MRPL12 inhibits ferroptosis in DKD mice

**Fig. 6:**
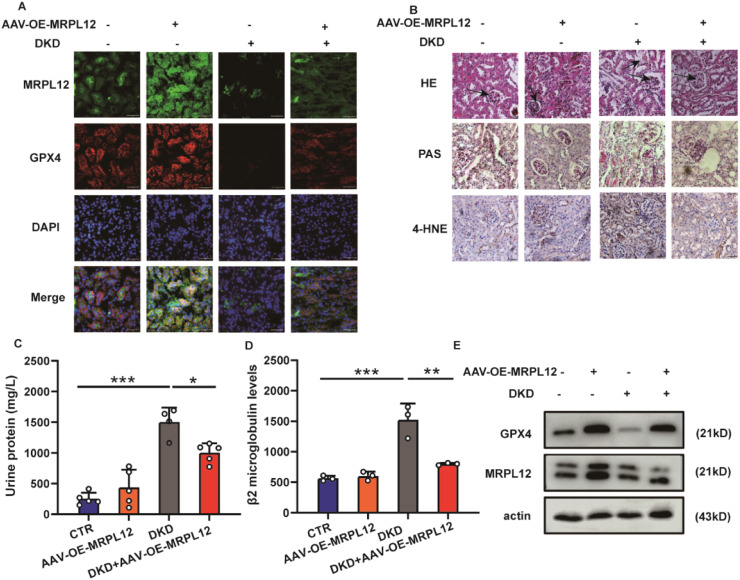
MRPL12 inhibited **ferroptosis in the DKD mice. (A)** Immunofluorescent analysis confirmed the overexpression of MRPL12 in renal tubular cells. Scale bars, 50 µm. (B) H&E staining (scale bars, 100 mm), PAS staining. (scale bars = 20 µm) and 4-HNE expression (scale bars = 100 μm) in the kidneys of mice. **(C)** Urine protein level. **(D)** β2 microglobulin levels. *P < 0.05, **P < 0.01, ***P < 0.001. **(E)** Western blotting analysis showing the expression of MRPL12, GPX4 in the kidney mice. *P < 0.05, **P < 0.01, ***P < 0.001.

Our findings from *in vitro* studies prompted further analysis of MRPL12 function in ferroptosis-associated pathological conditions. MRPL12 plays a key role in ferroptosis in DKD. Immunofluorescent analysis confirmed the overexpression of MRPL12 in renal tubular cells (Fig. 6A). As shown in Fig. 6B, 4-HNE was significantly increased in the DKD group, which was alleviated by MRPL12 overexpression. Furthermore, WB analysis showed that the expression of GPX4 and FTH was significantly decreased in the DKD group (Fig. 6E). Tubular injury in both groups was evaluated using H&E and PAS staining (Fig. 6B). We observed significant changes in MRPL12 expression between the control and DKD groups (Fig. 6E). We subsequently measured the urine protein and β2 microglobulin level which were found to be higher in the DKD group compared to the control group (Fig. 6C-6D). Collectively, these results suggest that MRPL12 overexpression inhibits ferroptosis in STZ-induced DKD, thereby potentially preventing renal injury.

## 7 Discussion

Renal tubular epithelial cell death is a hallmark feature of DKD. The high energy demand of these cells and their dependence on aerobic metabolism make them particularly vulnerable to diabetic conditions, which is why they are also the primary site of injury induced by hyperglycemia [18]. Ferroptosis- mediated cell death is critical in the development of DKD. Under high glucose condition, cells were treated with Fer-1, DFO, RSL3, Z-VAD-K, and Nec-1. As expected, Fer-1 and DFO alleviated high glucose-induced cell damage, whereas the caspase-3 or necroptosis inhibitor failed to show protective effects (Fig. 1A). Notably, Nec-1 reduced high glucose-mediated damage and attenuated inflammation in normal rat kidney tubular cells (NRK-52E) [19]. This discrepancy may stem from cell type-specific differences, including variations in metabolic signaling, pathway regulation, or transformation status, which could lead to distinct cellular responses to injury. Oral administration of an iron chelator alleviated oxidative stress and cell death in renal tissues of diabetic rats, and exerted a protective effect on their kidneys [20]. Our results indicate that high glucose condition in renal tubular epithelial cells leads to lipid peroxidation and an increase in iron levels, which indicates the occurrence of ferroptosis. This demonstrates that varying concentrations of iron ions are related to the progression of DKD [21].

Ferroptosis is a novel oxidatively regulated cell death driven by iron-dependent lipid peroxidation [22]. Organelles such as endoplasmic reticulum [[23], [24], [25]], lysosomes, and mitochondria play crucial roles in the ferroptosis process. A variety of metabolites are exchanged between mitochondria and the cytosol via voltage-dependent anion channels (VDACs) on the outer mitochondrial membrane (OMM). The ferroptosis inducer erastin induces the opening of VDAC2/3 [26]. The study by Gao et al. found that both cysteine deprivation (CD) and RSL3-induced ferroptosis lead to mitochondrial membrane potential (MMP) hyperpolarization, while mitochondrial uncoupler carbonyl cyanide m-chlorophenylhydrazone (CCCP) inhibits the process [26]. In mouse embryonic fibroblast (MEF) cells (Pfa1 cells), RSL3 treatment induced OMM rupture in a time-dependent manner [27]. Ferroptosis-mediated cell death caused changes in mitochondrial morphology and function [11]. Mitochondrial dysfunction has been identified as a critical event in the development of DKD. High glucose-treated HK-2 cells showed decreased ATP production, reduced mtDNA copy number, and increased ROS generation. At the molecular level, the expression of electron transport chain (ETC) complex subunits was downregulated. Furthermore, in RSL3-treated HK-2 cells, both mitochondrial dysfunction and alterations in ETC complex subunits were consistent with these findings, underscoring the pivotal role of mitochondria in DKD.

MRPL12 is a mitochondrial ribosomal protein reported to participate in metabolic [16,28] and neurodegenerative diseases [29]. A recent study found that MRPL12 associates with human mitochondrial RNA polymerase to activate transcription [30]. Our previous study showed that MRPL12 was significantly downregulated in high glucose-induced renal tubular epithelial cells. MRPL12 reduced oxidative phosphorylation (OXPHOS) and ATP production in high glucose-induced renal tubular epithelial cells [16,28]. In contrast, Rai et al. discovered increased MRPL12 levels in patients with diabetes with ischemic heart disease; MRPL12 overexpression did not increase OXPHOS and cellular ATP levels under hyperglycemic conditions. These finding imply that MRPL12 may play a compensatory role to maintain cellular homeostasis [28]. The role of MRPL12 in cell death remains uncertain. During AKI, MRPL12 expression is evidently decreased in TECs, and reduced MRPL12 results in decreased interaction between MRPL12 and adenine nucleotide translocase (ANT3), which subsequently causes ANT3 conformational change, abnormal mitochondrial permeability transition pore (mPTP) opening, and cell apoptosis. MRPL12 overexpression can protect HK-2 cells from abnormal mPTP opening and apoptosis during hypoxia/reoxygenation (H/R) [31].

Ferroptosis, a novel form of regulated cell death characterized by its unique lipid peroxidation-dependent mechanism, has emerged as a potential therapeutic target for various diseases, including cancer, neurodegenerative disorders, and ischemia-reperfusion injury. Activation of AMP-activated protein kinase (AMPK)/nuclear factor erythroid 2-related factor 2 (Nrf-2) pathway largely eliminates ferroptosis and ameliorates ferroptosis-associated renal ischemia-reperfusion injury [32]. Activation of the Nrf-2/HO-1/GPX4 signaling pathway protects against cerebral ischemia/reperfusion-induced blood-brain barrier damage by inhibiting ferroptosis [33]. In the present study, we found that MRPL12 is involved in regulating ferroptosis (Fig. 3). Likewise, MRPL12 overexpression protected HK-2 cells from lipid peroxidation during hyperglycemia and alleviated high glucose-induced renal injury (Fig. 6). In particular, we found that MRPL12 is involved in ferroptosis-mediated by GPX4, a key regulator of ferroptosis (Fig. 4). The protein encoded by *GPX4* belongs to the glutathione peroxidase family, members of which catalyze the reduction of hydrogen peroxide, organic hydroperoxides, and lipid hydroperoxides, and thereby protect cells against oxidative damage. Overexpression of MRPL12 increases GPX4 expression and thereby its antioxidant capacity (Fig. 4D). GPX4 has several transcription factors binding sites; Kruppel-like factor 2 (KLF2) is a transcription suppressor that inhibits GPX4 expression by combining with the 1057/−1046 region of the promoter [34]. Another negative transcriptional regulator of GPX4 is zinc finger E-box binding homeobox 1 (ZEB1). However, the critical signal pathways and regulation mechanism of GPX4 remain elusive.

Using bioinformatics methods, the shared transcription factors of GPX4 were predicted to include p53, VDR, and AR. p53 is involved in a variety of cellular processes including cell cycle arrest, DNA repair, metabolic alterations, antioxidant effects, anti-angiogenic effects, autophagy, senescence, and apoptosis. Recently, studies have established that p53 acts a novel regulator of ferroptosis [35,36]. More recently, p53 was found to act as a positive regulator of ferroptosis by inhibiting SLC7A11 expression (a specific light chain subunit of the cystine/glutamate antiporter) [37,38]. The potential binding site was verified using ChIP assay, and GPX4 was identified as a direct target gene of p53. These findings expand the mechanistic framework of p53 in controlling ferroptosis (Fig. 5). Notably, besides p53, other transcription factors (such as VDR and AR) were predicted to bind to the GPX-4 promoter and potentially activate its transcription (data available on request). While we focused on p53 due to its established role as a novel ferroptosis regulator, whether VDR and AR also contribute to GPX-4 regulation warrants further investigation. This is particularly relevant given that both molecules are known to be involved in numerous cellular processes, including inflammation, stress response, and tumorigenesis [39,40]. p53 plays a critical role in the pathogenesis of DKD, contributing to injury in glomerular mesangial cells and podocytes during DKD progression [41]. Our study reveals that p53 is also involved in renal tubular epithelial cell injury in DKD. In human cancers, p53 is degraded by high levels of the oncogenic E3 ubiquitin protein ligase MDM2. The suppression of MDM2-dependent proteasomal degradation of p53 provides a promising therapeutic strategy for cancer treatment. Notably, renal biopsy specimens from patients with DKD exhibited significantly reduced MDM2 gene expression in both glomerular and tubulointerstitial compartments [42]. We observed that high glucose stimulation significantly reduced MDM2 expression in HK-2 cells, and MDM2 exerted a protective effect, which is consistent with the findings of previous studies. MDM2 is reportedly associated with ferroptosis and regulates respiratory complex I activity [17].MRPL12 directly regulates the mtDNA-encoded subunits of the respiratory chain. Immunofluorescent staining and Co-IP were used to demonstrate that MRPL12 interacts with MDM2. In a high glucose environment, the binding between MRPL12 and MDM2 is reduced. In the present study, we demonstrated that MRPL12 is involved in ferroptosis and regulates GPX4 transcription via p53. Immunofluorescent staining and Co-IP were used to demonstrate that MRPL12 interacts with MDM2.

In summary, this study highlights that ferroptosis is involved in the development of DKD. During this process, MRPL12 regulates ferroptosis through the MDM2/p53/GPX4 pathway, and could be a link between ferroptosis and mitochondria. MRPL12 is potentially a novel therapeutic target for improving mitochondrial homeostasis and alleviating ferroptosis.

## Funding

This work was supported by the National Natural Science Foundation of China [81970427] and the Shandong Provincial Natural Science Foundation [ZR2025MS1234].

## CRediT authorship contribution statement

**Guoying Xia:** Writing – original draft, Formal analysis, Data curation. **Weiwei Zhang:** Writing – original draft, Formal analysis, Data curation. **Jinshi Liu:** Data curation. **Jian Yang:** Methodology, Data curation. **Qiuyao Jiang:** Supervision, Resources, Methodology. **Wei Xin:** Writing – review & editing, Supervision, Funding acquisition, Data curation.

## Declaration of competing interest

The authors have no conflict of interest to disclose.

## Data Availability

Data will be made available on request.

## Appendix Supplementary materials

Image, application 1
