# Supplementary figures and images for: MRPL12 regulates high glucose-induced ferroptosis in renal tubular epithelial cells via GPX4
Source: BBA Adv. 2026 Jun 23;10:100197. doi: 10.1016/j.bbadva.2026.100197 (PMC13351436; doi:10.1016/j.bbadva.2026.100197)

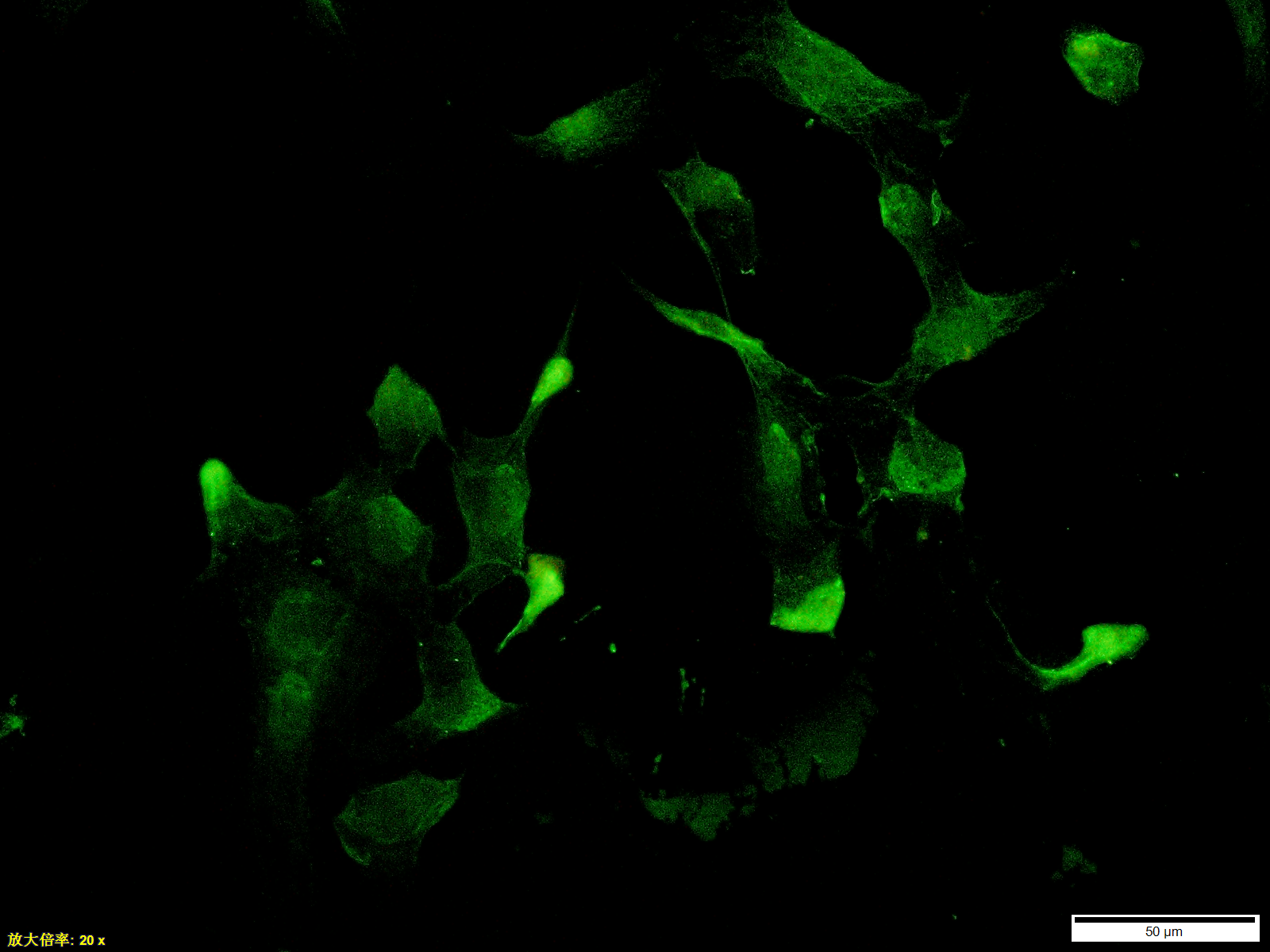

Supplement: Supplementary file 1 [file mmc1.zip › mmc1.tif]
